# Supplementary material for: A Humanized Yeast Phenomic Model of Deoxycytidine Kinase to Predict Genetic Buffering of Nucleoside Analog Cytotoxicity
Source: Genes (Basel). 2019 Sep 30;10(10):770. doi: 10.3390/genes10100770 (PMC6826991; doi:10.3390/genes10100770)
Supplement: Supplementary file 1 [file genes-10-00770-s001.zip › Additional_Files/Additional_File7_GOTermHeatmaps.zip!/Additional_File7_GOTermHeatmaps/maintenance_of_DNA_trinucleotide_repeats.pdf]

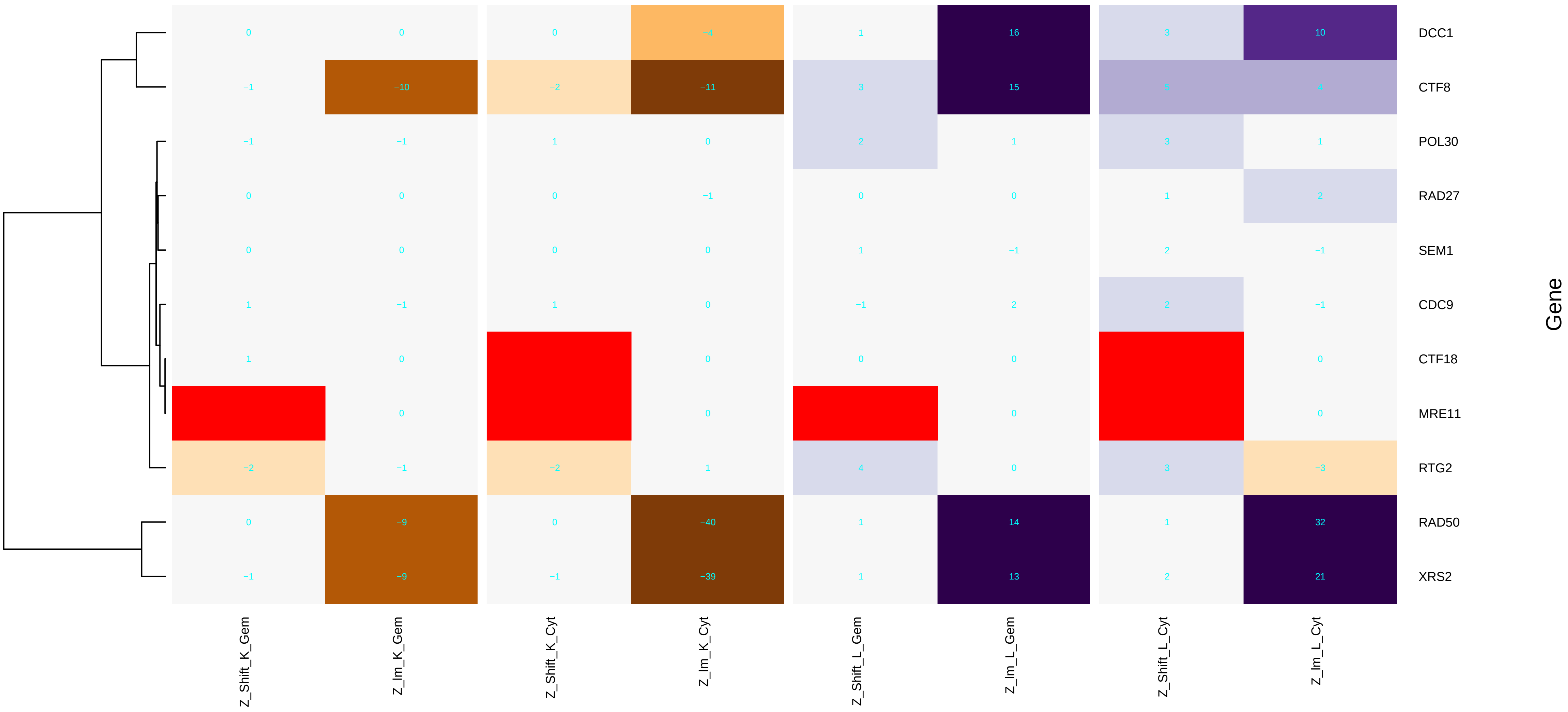

0 0 0 −4 1 16 3 10
DCC1
−1 −10 −2 −11 3 15 5 4
CTF8
−1 −1 1 0 2 1 3 1
POL30
0 0 0 −1 0 0 1 2
RAD27
0 0 0 0 1 −1 2 −1
SEM1
1 −1 1 0 −1 2 2 −1
CDC9
1 0 0 0 0 0
CTF18
0 0 0 0
MRE11
−2 −1 −2 1 4 0 3 −3
RTG2
0 −9 0 −40 1 14 1 32
RAD50
−1 −9 −1 −39 1 13 2 21
XRS2
Gene
Z_Shift_K_Gem
Z_Im_K_Gem
Z_Shift_K_Cyt
Z_Im_K_Cyt
Z_Shift_L_Gem
Z_Im_L_Gem
Z_Shift_L_Cyt
Z_Im_L_Cyt
